# Interleukin-31 and thymic stromal lymphopoietin expression in plasma and lymph node from Hodgkin lymphoma patients

**DOI:** 10.18632/oncotarget.19665

**Published:** 2017-07-28

**Authors:** Elisa Ferretti, Stefan Hohaus, Arianna Di Napoli, Beatrice Belmonte, Annarosa Cuccaro, Elisa Cupelli, Eugenio Galli, Vittoria Rufini, Gino Tripodi, Giulio Fraternali-Orcioni, Vito Pistoia, Anna Corcione

**Affiliations:** ^1^ Laboratory of Oncology, Istituto Giannina Gaslini, Genova, Italy; ^2^ Institute of Hematology, Catholic University of the Sacred Heart, Roma, Italy; ^3^ Department of Clinical and Molecular Medicine, Pathology Unit, Sant'Andrea Hospital, Sapienza University, Roma, Italy; ^4^ Tumor Immunology Unit, Department of Health Science, Human Pathology Section, University of Palermo, Palermo, Italy; ^5^ Institute of Nuclear Medicine, Catholic University of the Sacred Heart, Roma, Italy; ^6^ Immunohaematology and Transfusion Centre, Istituto Giannina Gaslini, Genova, Italy; ^7^ Unit of Pathology, IRCCS Azienda Ospedaliera Universitaria San Martino – IST – Istituto Nazionale per la Ricerca sul Cancro, Genova, Italy; ^8^ Immunology Research Area, Ospedale Pediatrico Bambino Gesù, Roma, Italy

**Keywords:** Hodgkin lymphoma, IL-31, TSLP, cytokine receptors, PET

## Abstract

Hodgkin Lymphoma (HL) is a tumor of B-cell origin characterized by Hodgkin and Reed-Stenberg (H/RS) cells embedded in an inflammatory tissue where numerous cytokines/chemokines contribute to shape the microenvironment, leading to the typical clinical symptoms.

We investigated: i) the expression of Interleukin-IL-31 (IL-31) and Thymic Stromal Lymphopoietin (TSLP), two Th2-related cytokines with tumor-promoting and pruritogenic functions, and of the respective receptors in HL invaded lymph nodes by flow cytometry, and ii) the potential association of IL-31/TSLP plasma concentrations with clinical characteristics by ELISA.

H/RS cells and the major immune cell types infiltrating HL lymph nodes expressed intracytoplasmic and surface IL-31/TSLP, and their receptors. A subgroup of patients showing at diagnosis elevated IL-31 and TSLP plasma levels had an International Prognostic Score>2, indicative of high risk of relapse, and a subsequent positive interim PET-scan, indicative of insufficient response to chemotherapy. No correlation was found between IL-31/TSLP plasma levels and overall or event-free survival.

In conclusion, IL-31/TSLP and their receptors are expressed in HL cells and in immune cells infiltrating affected lymph nodes, where both cytokines may contribute to local immune suppression. The clinical impact of IL-31 and TSLP plasma levels has to be further defined in larger patient cohorts.

## INTRODUCTION

Hodgkin Lymphoma (HL) is a B cell-derived malignancy characterized by low proportions of neoplastic mono-nucleated Hodgkin and multi-nucleated Reed-Stenberg (H/RS) cells in the invaded lymph nodes. HL is subdivided into classical (c) form, occurring in approximately 95% of cases, and nodular lymphocyte predominant (NLP) form (4-5% of cases), considered a different disease [1]. H/RS cells are embedded in a reactive microenvironment including CD4 T cells, B cells, macrophages, dendritic cells, eosinophils, fibroblasts, and basophils/mast cells [2]. This inflammatory microenvironment provides essential signals for H/RS cell survival [2, 3].

Although originating from germinal center (GC) or post-GC B cells [4], H/RS cells are characterized by down-regulation of B-cell markers and expression of CD15 and CD30 [5, 6]. Only a small proportion of HL (1-2%) originates from T cells [7].

Four histological subtypes of cHL have been identified based on HRS morphology and microenvironment composition: nodular sclerosis (80%), mixed cellularity (15%), lymphocyte rich and lymphocyte depleted [1].

The neoplastic tissue in Hodgkin lymphoma produces a wide spectrum of cytokines and chemokines that contribute to shape the microenvironment and lead to the typical clinical symptoms, as fever, night sweats, weight loss or pruritus.

Interleukin-31 (IL-31) is a cytokine related to the IL-6 family secreted by activated Th2 cells, monocytes, macrophages, dendritic cells and mast cells [8–10]. It signals through a heterodimeric receptor complex composed of the IL-31 Receptor Alpha (IL-31RA) and the Oncostatin M Receptor (OSMR) subunits [11–13]. Engagement of the IL-31R with IL-31 results in the activation of JAK1 and, to a minor extent, of JAK2 followed by activation of STAT1/3/5, MAPK, and PI3K signaling pathways [13–15]. It has been shown that IL-31 serum levels are increased in patients with cutaneous T cell lymphoma [16] and, more recently, our group has demonstrated that the IL-31/IL-31R axis promotes tumor growth in Follicular B cell lymphoma [16, 17].

Thymic Stromal Lymphopoietin (TSLP) is expressed by epithelial cells in the thymus, lung, intestine, skin, gut, and tonsil as well as by stromal cells and mast cells [18]. The high affinity TSLP receptor complex is composed of the IL-7R alpha chain/CD127 and the TSLP-specific Receptor component, TSLPR/Crlf2 [19]. The heterodimeric TSLP receptor activates, in addition to STAT5, STAT1/3, STAT4, and STAT6, as well as JAK1 and JAK2 [20]. The TSLP/TSLPR axis has been shown to promote tumor cell survival in both solid tumors and leukemia [21].

The involvement of IL-31 and TSLP as mediators of chronic pruritus in the pathogenesis of various skin diseases is clearly established [22–26]. Pruritus is observed in about 30% of patients with Hodgkin's lymphoma, more often in the nodular sclerosis type with mediastinal mass [27]. No information is available on the relationship among IL-31, TSLP and pruritus in HL patients. This latter issue has been here investigated by testing plasma levels of both cytokines and correlating them to pruritus and other clinical characteristics. In addition, we have investigated the expression of IL-31, TSLP and their receptors in invaded lymph nodes from HL patients in view of the tumor promoting role of these cytokines and the complete lack of information on this latter issue.

## RESULTS

### Expression of IL-31 and TSLP and the respective receptors in Hodgkin/Reed Sternberg cells and lymphoid cells populating the tumor microenvironment

**Figure 1:**
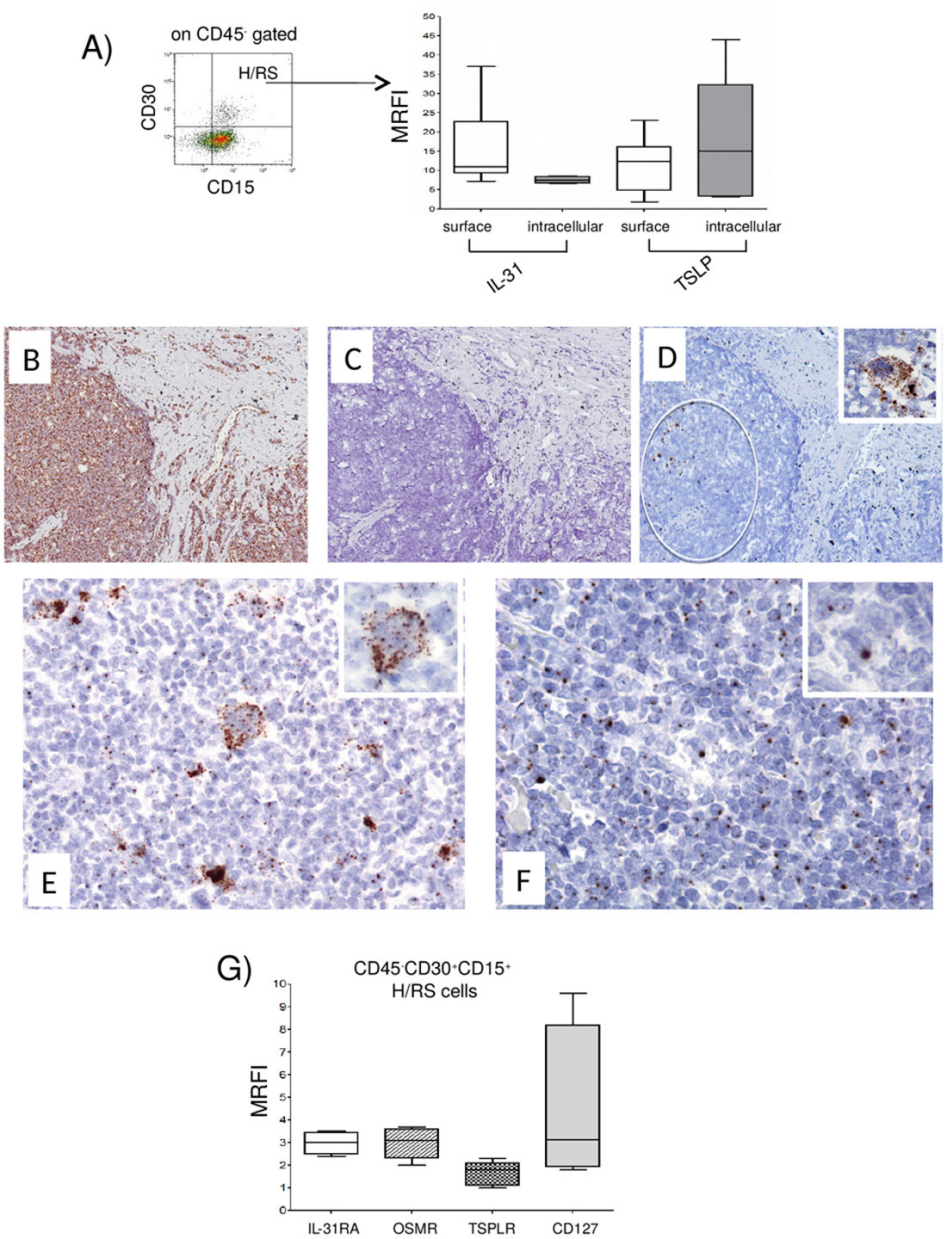
Expression of IL-31, TSLP and their receptors in H/RS cells **(A)** Left panel. A representative gating strategy for H/RS cells identified as CD45, CD30^+^, CD15^+^ cells. Right panel. IL-31/TSLP expression was tested by flow cytometry at surface and intracellular levels. Results are expressed in box plot as median MRFI, first and third quartiles, maximum and minimum values, from 10 different HL lymph node cell suspensions. **(B-F)** *In situ* hybridization for Ubiquitin **(B)**, dapB **(C)**, CD30 **(D)**, IL-31 **(E)** and TSLP **(F)** mRNA in cHL using the RNAscope technology **(B, C, D)** original magnification x100; E, F x200; insets x400). Ubiquitin mRNA was diffusely expressed (brown dots), whereas the bacterial dapB was completely negative. The CD30 probe hybridized with a proportion of the cells with H/RS morphology (circle and inset). Both IL-31 and TSLP mRNA were detected in the cytoplasm of H/RS cells (inset) and in some of the immune reactive cells present in the background. **(G)** IL-31RA/OSMR and TSLPR/CD127 chain receptor expression was analyzed by flow cytometry. Results are expressed in box plot as median MRFI, first and third quartiles, maximum and minimum values, from 7 different HL lymph node cell suspensions.

We have previously demonstrated that IL-31 is expressed on the surface membrane and in the cytoplasm of normal and Follicular Lymphoma B cells [17]. We therefore investigated by flow cytometry the surface and intracellular expression of both IL-31 and TSLP in lymph node biopsies from 10 HL patients. Cell suspensions isolated from invaded lymph nodes were multicolor stained with anti-CD30, -CD15, -CD45 mAbs in combination with the anti-IL-31 or -TSLP mAbs, and analyzed by flow cytometry after gating first on CD45^-^ cells, and then on CD30^+^, CD15^+^ H/RS cells (Figure 1A, left panel) [28].

In HL lymph nodes, H/RS cells, that ranged from 1 to 7%, median 3.4%, were found to express IL-31 and TSLP both at the cell surface (median MRFI IL-31= 11, range 7.2-37, n=10; median MRFI TSLP=12, range 2.0-23, n=8) (Figure 1A, right panel, first and third boxes, respectively, from the left) and intracellularly (median MRFI IL-31=7.5, range 6.6-8.6, n=6; median MRFI TSLP=15, range 3.2-44, n=6) (Figure 1A, right panel, second and fourth boxes, respectively, from the left).

To confirm the specificity of IL-31 and TSLP surface staining on H/RS cells, lymph node MNC cell suspensions were incubated in a solution at pH 2.5 for 10 minutes to elute surface-bound cytokines, washed and stained as above. Treatment at acidic pH causes detachment of soluble molecules non specifically adsorbed on the cell surface from the extracellular milieu, whereas it has no effect on endogenous surface molecules [29]. IL-31 and TSLP expression on the surface of H/RS cells was unaffected by treatment at acidic pH (not shown).

*In situ* hybridization with the RNAscope technology on paraffin sections from three HL lymph nodes using probes for IL-31 and TSLP showed clear punctate staining for both cytokines in cells with the morphology of H/RS cells. (Figure 1B-1F). Ubiquitin mRNA, tested as positive control, was diffusely expressed (brown dots), whereas the bacterial dapB, tested as negative control, was completely negative. The CD30 probe hybridized with a proportion of the cells with H/RS morphology (circle and inset). Both IL-31 and TSLP mRNAs were detected in the cytoplasm of H/RS cells (Figure 1E and 1F, insets) and in some of the immune reactive cells present in the background. In H/RS cells a high number of IL-31-positive dots/cell were evident (Figure 1E).

To investigate the expression of IL-31R and TSLPR in H/RS cells, cell suspensions from seven HL lymph nodes were stained with mAbs to IL-31RA, OSMR, TSLPR and CD127 and analyzed by flow cytometry gating on CD45^-^, CD30^+^, CD15^+^ cells as above. IL-31RA and OSMR, as well as TSLPR and CD127, were detected on H/RS cell surface (median MRFI IL-31RA=3.0, range 2.4-3.5; median MRFI OSMR=3.1, range 2.0-3.7; median MRFI TSLPR =1.8, range 1.0-2.3; median MRFI CD127=3.2, range 1.8-9.6) (Figure 1G, first to fourth boxes from the left, respectively).

Next, we addressed the expression of IL-31/TSLP and their receptors in the major cell types infiltrating the HL microenvironment. To this end, cell suspensions from seven HL lymph nodes and 7 reactive lymph nodes with follicular hyperplasia, tested as controls, were stained with B cell specific CD19 mAb, T helper cell specific CD4 mAb, or macrophage specific CD68 mAb, in combination with anti-IL-31 or -TSLP mAbs. Median values for CD19^+^ cells, CD4^+^ cells and CD68^+^ cells in HL lymph nodes were 39%, 62%, and 10%, respectively, while median values of the same cell populations for reactive lymph nodes were 39%, 47%, and 10%, respectively.

**Figure 2:**
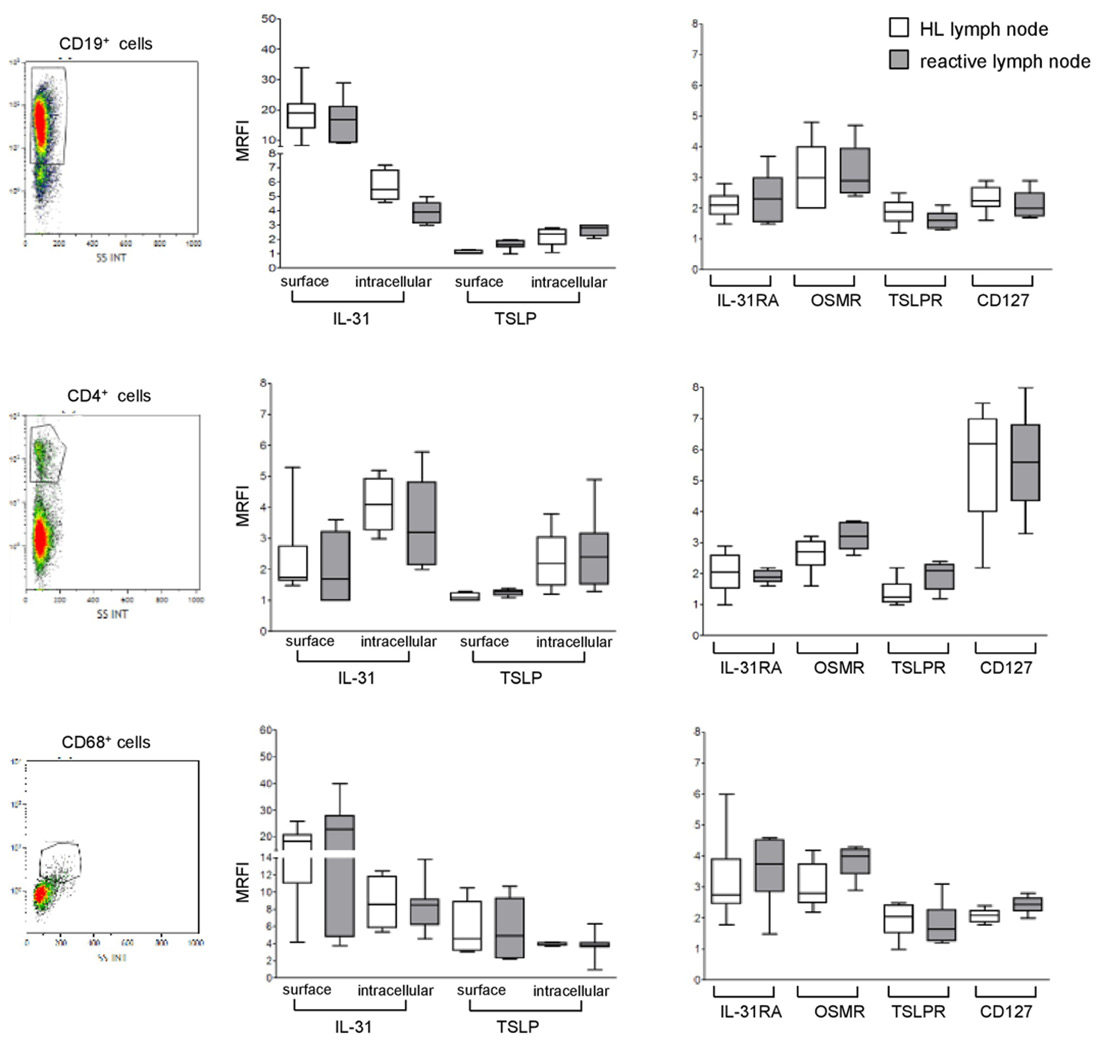
Expression of IL-31, TSLP and their receptors in lymphoid cells populating HL and reactive lymph node microenvironment Left panels. A representative gating strategy for B cells (CD19^+^), T cells (CD4^+^), and macrophages (CD68^+^). Middle panels. IL-31/TSLP expression was analyzed by flow cytometry at surface and intracellular levels on CD19^+^, CD4^+^, and CD68^+^ cells for both HL and reactive lymph nodes. Results are shown in box plot as median MRFI, first and third quartiles, maximum and minimum values, from 7 different HL and 7 reactive lymph node cell suspensions. Right panels. IL-31RA/OSMR and TSLPR/CD127 chain receptor expression was analyzed by flow cytometry on CD19^+^, CD4^+^ and CD68^+^ cells for both HL and reactive lymph nodes. Results are shown in box plot as median MRFI, first and third quartiles, maximum and minimum values, from 7 different HL and 7 reactive lymph node cell suspensions.

Consistent with our previous report [17], IL-31 was detected on the surface and in the intracellular compartment of CD19^+^ B cells from both HL and reactive lymph nodes (Figure 2, upper left panel). TSLP was found to be expressed in the same B cell suspensions in the intracellular compartment, whereas it was absent from the cell surface (Figure 2, upper left panel). Expression of IL-31 in CD4^+^ T cells was detected intracellularly and on the cell surface in both HL and reactive lymph nodes (Figure 2, middle left panel). TSLP was detected in the same cells intracellularly but not at the cell surface (Figure 2, middle left panel). Finally, IL-31 and TSLP were detected both at the cell surface and intracellularly in CD68^+^ macrophages from HL and reactive lymph nodes (Figure 2, lower left panel). Supplementary Table 1 reports in detail the median MRFI of IL-31 and TSLP detected in immune cell populations from both HL and reactive lymph nodes.

Surface staining for IL-31 and TSLP was superimposable in the latter cell fractions following pre-incubation at acidic pH (not shown). To confirm the flow cytometric experiments, we performed q-PCR analysis of mRNA in CD19^+^ B cells, CD4^+^ T cells and CD68^+^ macrophages isolated from tonsils, as well as in the L-428, HDLM-2, KM-H2 HL cell lines. The HeLa cell line was tested as positive control. As apparent, all of these cell types but the HL cell lines expressed the TSLP transcript (Supplementary Figure 1).

In additional experiments, we investigated the expression of IL-31R and TSLPR in the same cell suspensions from HL and reactive lymph nodes tested above. Both IL-31R and TSLPR were detected on CD19^+^ B cells (Figure 2, upper right panel). CD4 T cells expressed CD127, as well as IL-31RA, OSMR and TSLPR (Figure 2, middle right panel). Finally, macrophages expressed IL-31RA and OSMR, as well as TSLPR and CD127 (Figure 2, lower right panel). Median MRFI values for IL-31/TSLP receptors for each immune cell populations in HL and reactive lymph nodes are reported in Supplementary Table 2.

As apparent from both Figure 2 and Supplementary Tables 1 and 2, no differences in the expression of IL-31, TSLP and the respective receptors were found between immune cells present in HL and those present in reactive lymph nodes.

Finally, we investigated the expression by flow cytometry of IL-31, TSLP and the respective receptor chains in HDLM-2, L-428, and KM-H2 HL cell lines. None of the cell lines tested expressed the two cytokines or their receptors (not shown).

### IL-31 and TSLP plasma levels in patients with Hodgkin lymphoma

**Figure 3:**
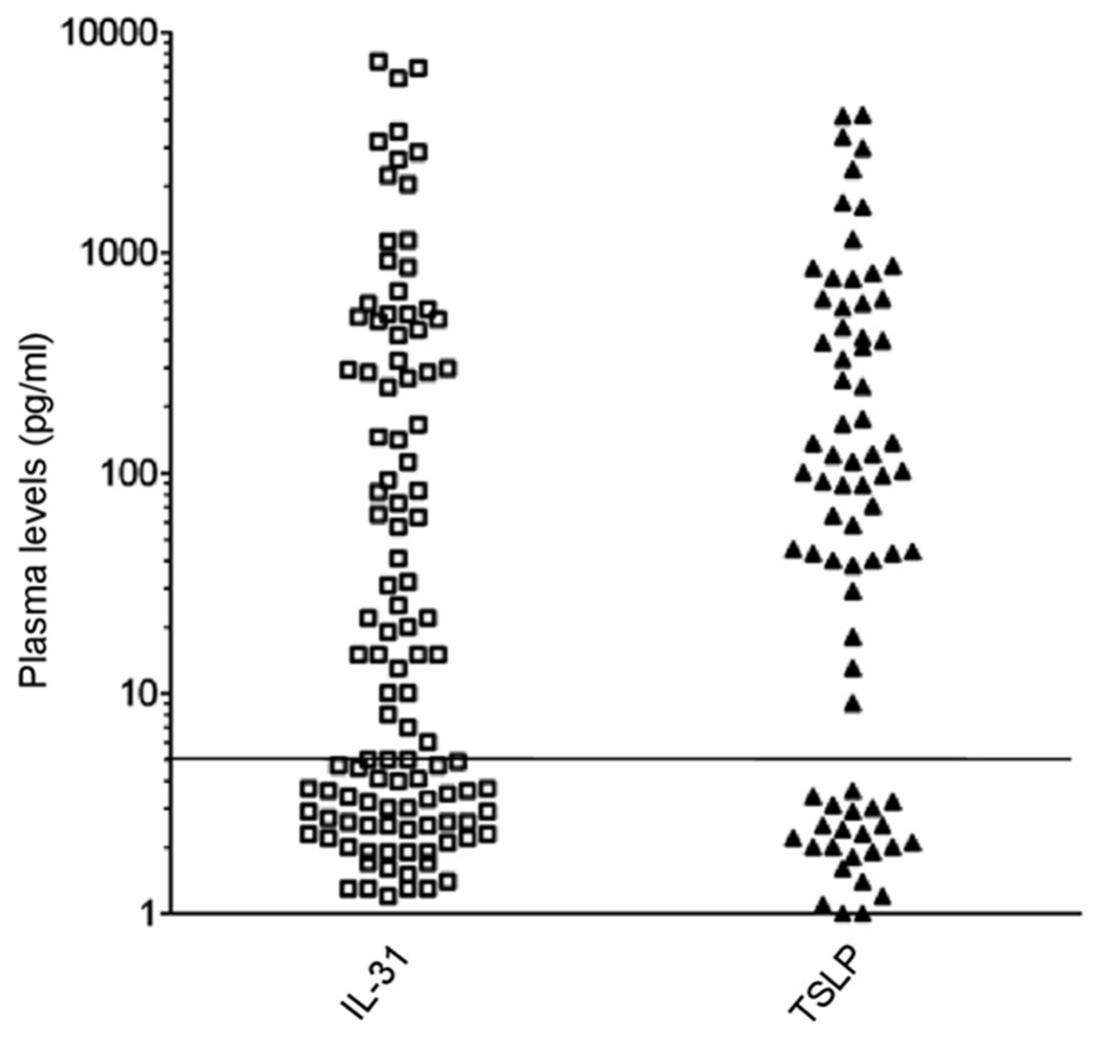
sIL-31/TSLP plasma levels in HL patients sIL-31/TSLP levels were assayed by ELISA (threshold of detection 5 pg/ml). sIL-31 was detected in 65/109 patients, sTSLP in 52/75 patients. The horizontal line separates positive from negative results.

We next analyzed soluble (s)IL-31 and sTSLP levels in plasma samples from HL patients at diagnosis and from healthy controls. sIL-31 was detected in 65/109 (60%) patients (Figure 3) with a wide range from 5 to 7937 pg/ml and a median of 245 pg/ml. sTSLP was detected in 52/75 (69%) patients tested (Figure 3) with a range from 9 to 4209 pg/ml, and a median of 171 pg/ml.

sIL-31 and sTSLP levels in HL patients did not differ from the levels detected in a group of 84 age-matched controls. Thus, sIL-31 was detected in 42/84 (50%) healthy controls ranging from 8 to 7714 pg/ml (median of 281 pg/ml), while sTSLP was detected in 32/59 (54%) healthy controls ranging from 10 to 2849 pg/ml, with a median of 134 pg/ml (Supplementary Figure 2). A highly significant correlation between sIL-31 and sTSLP concentrations was observed in both HL patients and healthy controls (*P*<0.0001), suggesting a coordinate production of the two cytokines, possibly operated by the same cells.

We next analyzed for associations between cytokine levels and patient clinical characteristics. HL is often associated with pruritus [27], present in 47% of our patients. Pruritus was defined as: i) intense, if widespread and associated with secondary cutaneous lesions due to scratching and/or need for anti-histamines; and ii) mild, when only localized. sIL-31 or sTSLP were not found to be associated to the presence or degree of itching in the HL patients studied (n=24 no pruritus, n=14 mild, n=21 intense) (Supplementary Figure 3A and 3B, respectively).

The International Prognostic Score (IPS) is the most widely used risk stratification index for HL that incorporates seven clinical parameters independently associated with poor outcome [30]. IPS from 0 to 2 identify low-risk patients, while IPS values>2 are detected in high-risk patients. Significantly higher levels of sIL-31 and sTSLP (*P*=0.002, n=34/72, median 182 pg/ml for IL-31, *P*=0.03, n=25/48, median 167 pg/ml for TSLP) were detected in HL patients with an IPS >2 compared to those with IPS 0-2. Among the clinical parameters included in IPS, WBC count >15x10^3^/ml was found to be significantly associated with high sIL-31 and sTSLP levels in HL patients studied (*P*=0.01, n=19/89 for IL-31; *P*=0.02, n=15/59 for TSLP). No correlation with other patient characteristics was identified. Tables 1 and 2 show in detail the results on the associations between clinical characteristics and sIL-31 or sTSLP, respectively.

**Table 1:** Associations between clinical patient characteristics at diagnosis and IL-31 plasma levels in HL patients

		*Variable*	*Number*	*IL-31 level* *median* *(pg/ml)*	*P*
**IPS parameters**	**Age, years** (n=109)	< 45 >45	75 34	10 14	0.9
	**Gender** (n=109)	Female Male	53 56	15 5	0.4
	**Stage** (n=109)	I-III IV	80 29	5 83	0.1
	**White blood cell count** (n=108)	≤ 15 x 10^3^/ml > 15 x 10^3^/ml	89 19	5 294	**0.01**
	**Lymphocyte count** (n=105)	≥ 600/ml < 600/ml	93 12	7 18	0.4
	**Hemoglobin level** (n=108)	≥ 10.5 g/dl < 10.5 g/dl	89 19	7 25	0.2
	**Albumin level** (n=105)	≥ 40 g/l < 40 g/l	55 50	5 21	0.1
**IPS** (n=106)		0-2 3-5	72 34	5 182	**0.002**
**Histology subtype** (n=109)		cHL Nodular sclerosis cHL Mixed cellularity cHL lymphocyte rich cHL NOS NLPHL	86 1 4 15 3	15 7 9 13 15	0.5
**B-symptoms** (n=109)		Absent Present	68 41	5 22	0.2

**Table 2:** Associations between clinical patient characteristics at diagnosis and TSLP plasma levels in HL patients

		*Variable*	*Number*	*TSLP level* *median* *(pg/ml)*	*P*
**IPS parameters**	**Age, years** (n=75)	< 45 >45	52 23	80 102	0.9
	**Gender** (n=75)	Female Male	39 36	58 101	0.3
	**Stage** (n=75)	I-III IV	55 20	71 94	0.6
	**White blood cell count** (n=74)	≤ 15 x 10^3^/ml > 15 x 10^3^/ml	59 15	45 246	**0.02**
	**Lymphocyte count** (n=71)	≥ 600/ml < 600/ml	63 8	71 112	0.7
	**Hemoglobin level** (n=74)	≥ 10.5 g/dl < 10.5 g/dl	60 14	88 80	1.0
	**Albumin level** (n=73)	≥ 40 g/l < 40 g/l	37 36	44 117	0.3
**IPS** (n=73)		0-2 3-5	48 25	44 167	**0.03**
**Histology subtype** (n=75)		cHL Nodular sclerosis cHL Mixed cellularity cHL NOS NLPHL	61 1 10 3	117 71 63 582	0.9
**B-symptoms** (n=75)		Absent Present	47 28	91 52	0.2

**Figure 4:**
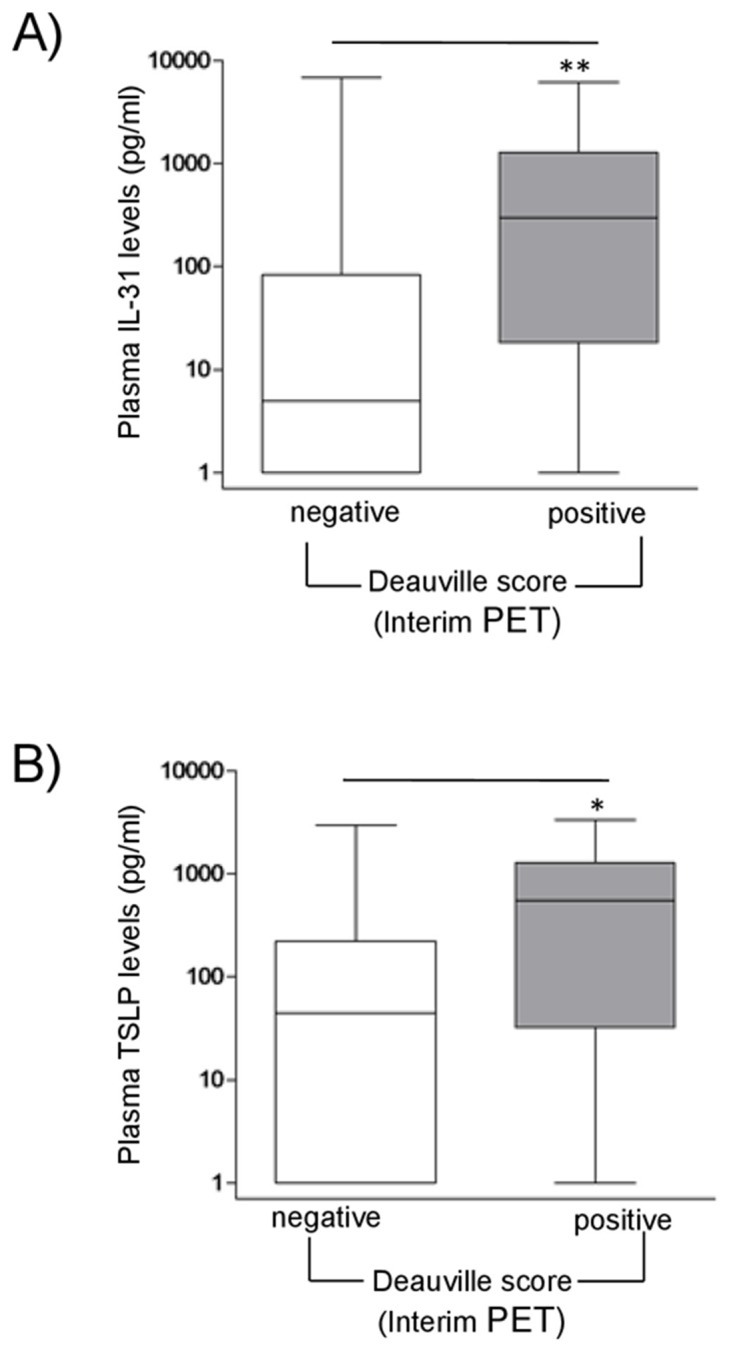
Correlations between sIL-31/sTSLP and Interim Pet in HL patients sIL-31 **(A)** and sTSLP **(B)** levels (pg/ml) in HL patient plasma were correlated with interim PET scan. PET images were classified according to the Deauville score (1-3 negative; 4-5 positive). HL patients with a positive score had significant higher median levels of sIL-31 and sTSLP than patients with negative score (^**^P=0.01, n=13/84 and ^*^P=0.05, n=10/63, respectively).

Early response following 2 cycles of chemotherapy is evaluated using (^18^F)-fluordeoxyglucose (FDG)-position emission tomography (PET) (interim PET), which represents the strongest predictor to distinguish high-risk from low-risk HL patients [31]. PET images are scored according to the Deauville classification system [31]. FDG uptakes in residual tissue below the uptake in the liver were considered as negative (Deauville score 1-3), while uptake higher than the liver background was considered as positive (Deauville score 4-5) [31, 32]. Interestingly, HL patients with a positive interim PET-scan, indicative of high risk of relapse, had significantly higher levels of sIL-31 (*P*=0.01, n=13/84, median=298 pg/ml) and sTSLP (*P*=0.05, n=10/63, median 548 pg/ml) at diagnosis compared to patients with a negative interim PET-scan (Figure 4A and 4B, respectively).

With a median observation of 32 months, 30/109 patients experienced an event. This translated into a 3-year probability of event-free survival (EFS) of 75% (95% C.I., 65-82%). Patients with sIL-31and sTSLP levels higher than the median did not differ in their prognosis from patients with levels below the median. Likewise, HL patients were analyzed for overall survival (OS): the 3-year OS was 90% ( 95% C.I., 82-95%) without any difference between patients with sIL-31/TSLP levels higher or lower than the median (data not shown).

## DISCUSSION

In this study, we demonstrate that H/RS cells express intracytoplasmic and surface IL-31 and TSLP, as well as the respective receptors. B cells, macrophages and CD4^+^ T cells infiltrating HL lymph nodes showed surface and cytoplasmic expression of IL-31, while TSLP was detected on the cell surface of macrophages and in the cytoplasm of B cells, CD4^+^ T cells and macrophages. IL-31/TSLP and their receptors were expressed with superimposable profiles in the same immune cell fractions from reactive lymph nodes. The finding that both malignant and immune cells in the HL lymph node microenvironment expressed IL-31 and TSLP and the respective receptors suggests that numerous paracrine and/or autocrine interactions may take place *in vivo*. These cytokines may contribute to cell-to-cell interactions by shedding of soluble forms from the surface membrane or release of the cytoplasmic forms, and ii) direct contact between surface bound cytokine(s) on a cell and the respective receptor(s) on an adjacent cell. In this respect, we have previously demonstrated that IL-31 is not released in soluble form by Follicular Lymphoma B cells, but shed in microvesicles that serve as intercellular messengers. This mechanism, that has not been reported for TSLP and may operate also in H/RS cells [33] could not be investigated due to the paucity of the latter cells in affected lymph nodes.

Plenty cytokines/chemokines released by H/RS cells shape the tumor microenvironment. Thus, for example, IL-5, CCL5, CCL28 attract eosinophils [34–36]; CCL5 attract mast cells, [37] IL-8 neutrophils [34], CCL5, CCL17, CCL22 Th2 cells, and CCL20 T reg cells [38–41]. Although the HL microenvironment is considered as Th2 polarized, this may be an oversimplification, since a recent study demonstrates that T cells infiltrating HL lymph nodes express Th1-type chemokine receptors, cytokines and transcription factors [42].

A question raised by this study is how IL-31 and TSLP can modulate the HL lymph node microenvironment to support tumor growth. In atopic dermatitis, IL-31 induces chemotaxis, Ca^2+^ mobilization, release of reactive oxygen species, surface expression of adhesion molecules and CCL26 in eosinophils, which in turn release IL-31, contributing to the maintenance of the inflammatory infiltrate [43, 44]. TSLP promotes directly commitment of human bone marrow hematopoietic progenitors to the eosinophil/basophil lineage and elicits mature basophil responses in the periphery [45, 46]. In addition, TSLP can enhance eosinophil survival, up-regulate surface expression of adhesion molecules and induce the release of inflammatory cytokines and chemokines from human eosinophils [47]. Finally, TSLP amplifies M2 macrophage polarization [48, 49]. Since eosinophils and, at a lower extent, basophils/mast cells are important components of the HL lymph node infiltrate, it is conceivable that IL-31 and/or TSLP contribute to the recruitment, survival and activation of these cell types and, more in general, polarize immune responses towards a tumor promoting functional state. A note of caution in the discussion of the potential mechanisms whereby IL-31 and TSLP can shape the HL microenvironment comes from previous studies showing the existence of different IL-31RA isoforms, some of which are devoid of signalling activity [17, 50]. Furthermore, two dominant isoforms of IL-7R alpha chain/CD127 coding for membrane-bound or soluble IL-7R alpha, respectively, have been identified [51], and two isoforms of TSLP with completely different functions have been reported [52].

IL-31 and TSLP are two Th2-related cytokines that promote itch in atopic skin diseases by activating cutaneous somatosensory neurons, either directly or indirectly through stimulation of immune cells [53]. However, no association was detected between concentrations of sIL-31 or sTSLP and presence or degree of itching in a cohort of HL patients at diagnosis. In contrast, we found an association between high plasma levels of both cytokines and increased number of circulating WBC, in particular neutrophils. In this respect, IL-31was found to stimulate in a mouse model the survival of myeloid progenitor cells [54], raising the possibility that in HL patients IL-31 induces neutrophilia, possibly in concert with other cytokines that have myelopoietic activity as CXCL8 [55], Since the WBC count is a prognostic factor included in the IPS score, both IL-31 and TSLP levels were higher in patients with an IPS>2, indicative of high risk disease.

Finally, patients with a positive interim PET-scan, usually indicative of insufficient response to chemotherapy [31], had higher levels of sIL-31 and sTSLP at diagnosis. The association of positive interim PET-scan and high cytokine plasma levels may simply reflect differences in persistence of an inflammatory microenvironment in the scanned lymph nodes at the time of interim PET without truly reflecting disease activity, since we failed to identify any correlation between sIL-31 or sTSLP plasma levels and event-free or overall survival in our patient cohort. In addition, heterogeneity in therapeutic protocols may have influenced the correlations between plasma cytokines, interim PET scan and outcome. Further studies in a larger patient group are needed to better define the clinical impact of IL-31 and TSLP plasma levels.

## MATERIALS AND METHODS

### Patients and controls

The analysis included 109 patients (56 males and 53 females, 34 of whom >45 years and 75 <45 years), diagnosed with cHL (106 patients) and NLPHL (3 patients) at the Department of Hematology of the Catholic University of Rome, Italy. Six of the patients studied had an age range from 15 to 18 years. Diagnosis of HL was established according to the criteria of the World Health Organization (WHO) classification [1]. cHL histology subtype was the following: 86 Nodular sclerosis, 1 Mixed cellularity, 4 Lymphocyte-rich and 15 that could not be classified into any subtype and are referred to as “not otherwise specified”. Eighty-four healthy donors (45 males and 39 females, 36 of whom >45 years and 48 <45 years,) were recruited from the Division of Immunohematology and Transfusion Centre, Giannina Gaslini Institute, Genoa, Italy. The study was approved by the Institutional Review Board of the Catholic University (P/416/CE/2010) and the Institutional Review Board of the Istituto Giannina Gaslini, Genova, Italy on October 27^th^, 2005. Informed consent was obtained from both patients and healthy donors according with the Declaration of Helsinki.

### Cell isolation

Invaded lymph nodes from 10 HL patients (6 males and 4 females, 3 patients >45 years, 7 patients<45 years) and 7 reactive lymph nodes biopsied for diagnostic purposes, were obtained from the San Martino Hospital-Istituto Scientifico Tumori Biobank (Genova, Italy) and from the Sant'Andrea Hospital (Roma, Italy) (Institutional Review Board n°168/2003). Lymph node mononuclear cells (MNCs) were isolated after a gentle mince and cryopreserved in a freezing solution composed of 50% RPMI 1640 (Sigma Chemical Co., St. Louis, MO), 40% fetal bovine serum (FBS) (Sigma), and 10% DMSO (Sigma). Cells were kept in liquid nitrogen until tested.

The human HDML-2, L-428, KM-H2cell lines, established from HL patients, were provided five months ago by DSMZ (Braunschweig, Germania) that certifies their origin. These cell lines were cultured in RPMI 1640 medium (Sigma Saint Louis, Missouri, USA) supplemented with 10% fetal bovine serum (FBS) (Sigma).

### Antibodies for flow cytometry

The monoclonal Antibodies (mAbs) used throughout the study were the following: Phycoerythrin (PE)-conjugated anti-human IL-31RA from R&D System (Minneapolis, MN, USA); phycocyanin (PC)7-CD19; PE-anti-human OSMR, PC7-CD4, Fluorescein Isothiocyanate (FITC)-CD68, PE-CD127, PE-anti-human TSLPR from eBioscience (San Diego, CA). APC-conjugated anti-human IL-31 and unconjugated anti-human TSLP were from Lifespan Biosciences (Seattle, USA) and Abcam, (Cambridge, UK), respectively. Cells were stained with fluorochrome conjugated or unconjugated antibodies followed by secondary reagents. Isotype and fluorochrome matched antibodies were tested as controls. Cells were run on a Gallios instrument (Beckman Coulter, Brea, CA, USA) and data were analyzed using the Kaluza software (Beckman Coulter). On average 30000/40000 events were acquired. Results were expressed as Mean Relative Fluorescence Intensity (MRFI), calculated as follows: fluorescence intensity obtained with specific mAb/fluorescence intensity obtained with irrelevant isotype-matched mAb. For intracellular cytokine staining cells were fixed, permeabilized using cytofix and perm kit (Becton Dickinson, New Jersey, USA) and stained with anti-IL-31and -TSLP or isotype-control mAbs and analyzed as above.

For some experiments, MNCs from three HL and three reactive lymph nodes were suspended in 0.5M NaCl and 0.2M acetic acid (pH 2.5) and held at 4°C for 10 minutes to elute surface-bound cytokines. Cells were then washed twice with PBS and subsequently stained as above.

### RNAscope

The RNAscope assay was applied to lymph node paraffin sections from three HL patients using probes to IL-31 and TSLP, as previously described [56]. Briefly, formalin fixed, paraffin embedded (FFPE) tissue sections 2 μm thick were deparaffinized in xylene and then hydrated in an ethanol series. Hybridization was performed with the negative control probe dapB, the positive control probe Probe-Hs-Ubiquitin, and the target probes Probe-Hs-IL-31 and Probe-Hs-TSLP. The preamplifier, amplifier, label probe, and chromogenic detection procedures were performed according to the manufacturer’s instructions (RNAscope^®^ 2.0 HD Reagent Kit, Advanced Cell Diagnostics, Hayward, CA, USA).

### RT-PCR

Total RNA was isolated using the RNeasy kit (Qiagen, Milano, Italy) according to the manufacturer’s instructions. RNA was assessed for integrity by gel electrophoresis and quantified by spectrophotometry (Nanodrop Products, Wilmington, DE). One μg of total RNA was reverse transcribed using the High Capacity cDNA Reverse Transcription kit (Life Technologies, Monza, Italy), according to manufacturer’s instructions. The primer sequences for human TSLP and GAPDH mRNA and the relative PCR conditions were as described [57]. All the primers were purchased from TIB Molbiol (TIB MolBiolS.r.L., Genova, Italy). The amplified products were visualized by electrophoresis on a 2% agarose gels. Images were analyzed by scanning using the VersaDoc instrument (BioRad Laboratories, Segrate, Italy). PCR reactions for each sample were performed at least twice.

### ELISA

Plasma samples from HL patients, collected at diagnosis prior to treatment start, and from healthy controls were tested for IL-31 (n=109 and n=84, respectively) and TSLP (n=75 and n=59, respectively) by ELISA (RayBiotech, Inc., Parkway Lane, Norcross, GA, USA). The sensitivity threshold for both the immunoenzymatic assays was lower than 5 pg/ml.

### Statistical analysis

Data were reported in box plot in terms of medians, first and third quartiles, minimun and maximum values. The Mann-Whitney U test was used to compare quantitative variables between two groups of observation with 99% confidence interval (GraphPad Prism 3). The Spearman test was used for the correlation between IL-31 and TSLP levels. All statistical tests were two tailed and a *P* value lower than 0.05 was considered statistically significant. Statistical analyses were performed using Graph Pad Prism 5software.

## SUPPLEMENTARY MATERIALS FIGURES AND TABLES



## Abbreviations

(HL): Hodgkin lymphoma
(HRS): Hodgkin Reed-Stenberg
(cHL): classical
(NLPHL): nodular lymphocyte predominant HL
(GC): germinal center
(IL): interleukin
(IL-31RA): IL-31 receptor alpha
(OSMR): oncostatin M receptor
(TSLP): thymic stromal lymphopoietin
(s): soluble
(IPS): International Prognostic Score
(FDG): (^18^F)-fluordeoxyglucose
(PET) (interim PET): position emission tomography
(EFS): event-free survival
(OS): overall survival
(WHO): World Health Organization
(FBS): fetal bovine serum
(mAbs): monoclonal antibodies
(PE): phycoerythrin
(FITC): fluorescein isothiocyanate
(PC): phycocyanin
(MRFI): mean relative fluorescence intensity
(FFPE): formalin fixed paraffin embedded
