# Supplementary material for: Interleukin-31 and thymic stromal lymphopoietin expression in plasma and lymph node from Hodgkin lymphoma patients
Source: Oncotarget. 2017 Jul 28;8(49):85263–75. doi: 10.18632/oncotarget.19665 (PMC5689608; doi:10.18632/oncotarget.19665)
Supplement: Supplementary file 1 [file oncotarget-08-85263-s001.pdf]

Interleukin-31 and thymic stromal lymphopoietin expression in plasma and lymph node from Hodgkin lymphoma patients

SUPPLEMENTARY MATERIALS

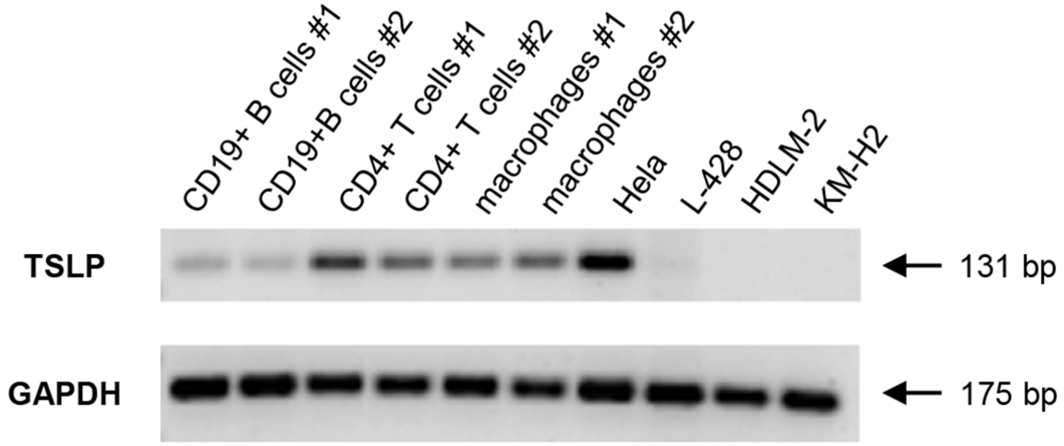

Supplementary Figure 1: mRNA expression of the TSLP and GAPDH genes. mRNA was detected by RT-PCR in CD19+B cells, CD4+T cells, and in three HL cell lines (L-428, HDLM-2, KM-H2). The HeLa cell line was tested as positive control.

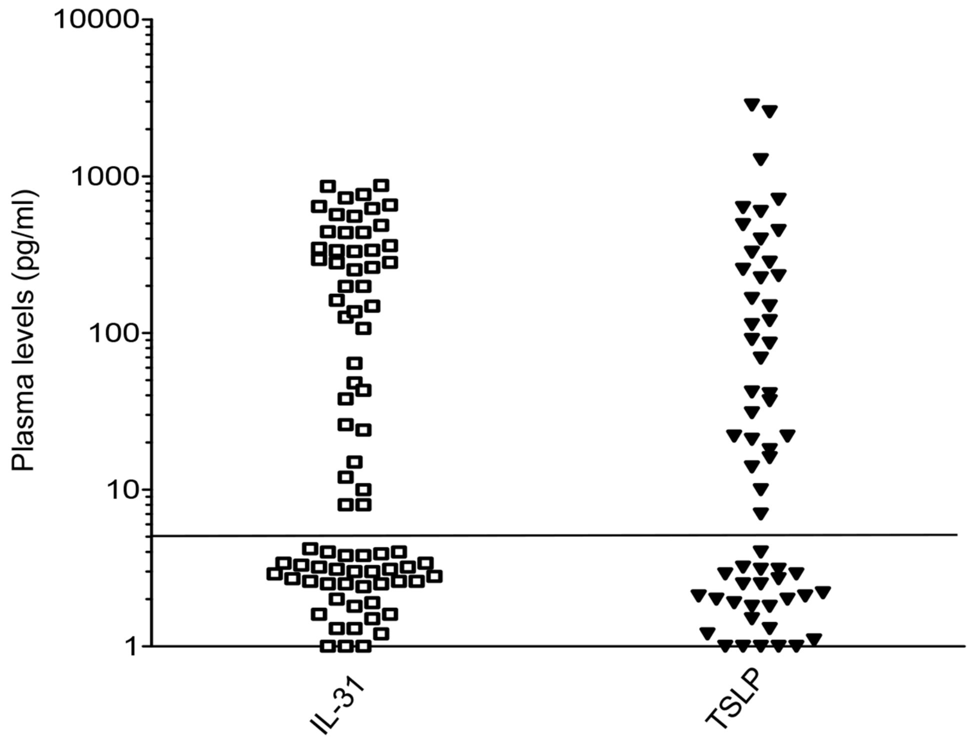

Supplementary Figure 2: IL-31/TSLP plasma levels in healthy controls. sIL-31/TSLP levels were assayed by ELISA (threshold of detection 5 pg/ml).sIL-31 was detected in 42/84 donors, sTSLP in 32/59 donors. The horizontal line separates positive from negative results.

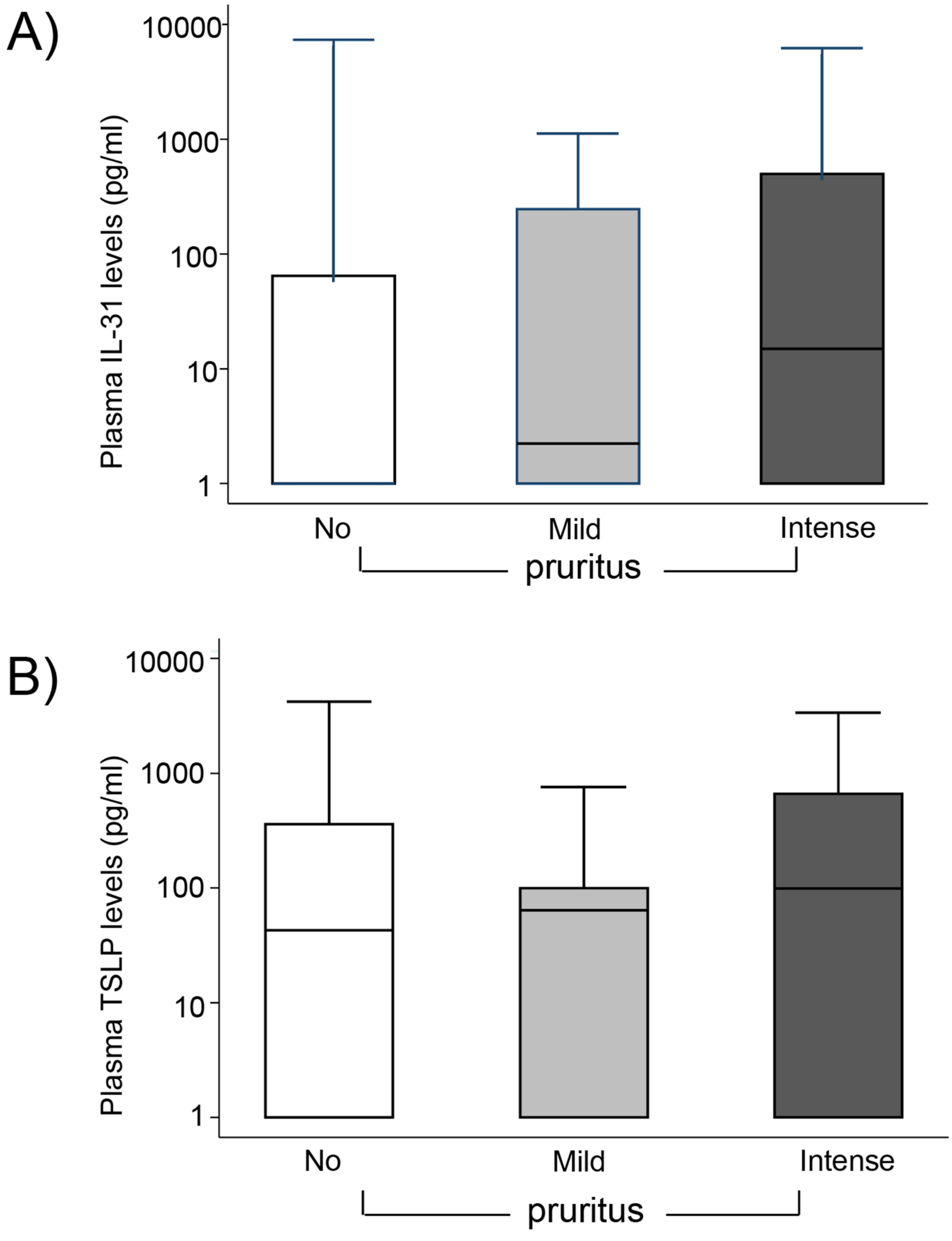

Supplementary Figure 3: IL-31 and TSLP plasma levels and pruritus in HL patients. sIL-31 **(A)** and sTSLP **(B)** plasma levels (pg/ml) in 69 HL patients were correlated with pruritus defined as: none, mild, and intense. Results are expressed in box plot as median levels, first and third quartiles, maximum and minimum values.

Supplementary Table 1: IL-31 and TSLP expression

	HL lymph node				Reactive lymph node			
	**IL-31 surface**	**IL-31 intracellular**	**TSLP surface**	**TSLP intracellular**	**IL-31 surface**	**IL-31 intracellular**	**TSLP surface**	**TSLP intracellular**
**Median MRFI on CD19+ cells**	**19** (8.3-34)	**5.5** (4.6-7.2)	**1.1** (1.0-1.3)	**2.4** (1.1-2.8)	**16.8** (9-28.9)	**3.9** (3-5)	**1.6** (1-2)	**2.8** (2.1-3)
**Median MRFI on CD4+ cells**	**1.7** (1.5-5.3)	**4.1** (3.0-5.2)	**1.1** (1.0-1.3)	**2.2** (1.2-3.8)	**1.7** (1-3.6)	**3.2** (2-5.8)	**1.3** (1.1-1.4)	**2.4** (1.3-4.9)
**Median MRFI on CD68+ cells**	**18.5** (4.2-26)	**8.6** (5.4-12.5)	**4.6** (3.1-10.5)	**3.9** (3.4-4.2)	**23** (3.8-40)	**8.5** (4.6-13.8)	**4.9** (2.2-10.7)	**3.8** (1-6.3)

Median MRFI of IL-31 and TSLP detected in immune cell populations from both HL and control reactive lymph nodes.

Supplementary Table 2: IL-31 and TSLP receptors expression

	HL lymph node				Reactive lymph node			
	**IL-31RA**	**OSMR**	**TSLPR**	**CD127**	**IL-31RA**	**OSMR**	**TSLPR**	**CD127**
**Median MRFI on CD19+ cells**	**2.1** (1.5-2.8)	**3** (2-4.8)	**1.9** (1.2-2.5)	**2.2** (1.6-2.9)	**2.3** (1.5-3.7)	**3.2** (2.4-4.7)	**1.6** (1.3–2.1)	**2.1** (1.7–2.9)
**Median MRFI on CD4+ cells**	**2** (1-2.9)	**2.7** (1.6-3.2)	**1.2** (1-2.2)	**6.2** (2.2-7.5)	**1.9** (2.6-2.2)	**3.2** (2.6-3.7)	**2.1** (1.2-2.4)	**5.6** (3.3-8)
**Median MRFI on CD68+ cells**	**2.7** (1.8-6)	**2.8** (2.2-4.2)	**2** (1-2.5)	**2.1** (1.8-2.4)	**3.4** (1.5-4.6)	**4** (2.9-3)	**1.6** (1.2-3.1)	**2.4** (2-2.8)

Median MRFI of IL-31R and TSLPR detected in immune cell populations from both HL and control reactive lymph nodes.
